# Painless Parturition

**Published:** 1863-05

**Authors:** 


### PAINLESS PARTURITION.

Dr. George Smith, of Madras, communicated to the Obstetrical Society of Edinburgh the following example of this:

"Some years ago I was engaged to attend an English lady during her approaching confinement, and was startled one day by a hasty summous, coupled with the information that the child had been suddenly born without warning of any kind. On reaching my patient's residence, I found that the child had been born about ten minutes, and that it was still lying, with the umbilical cord uncut, close to the mother's body. The native female servant, at the lady's order, had left the child untouched, merely raising the bedclothes a little to permit the free access of air for the purpose of respiration.

"On inquiry, the lady informed me that she had been for some time expecting her confinement daily; that the previous night she had felt as usual; but that she had had occasion to rise frequently to attend upon her sick child, and that she had got up as usual about half-past five A. M., feeling well, and having no indication of the near approach of labor. Further, that during the forenoon she had walked down a long flight of steps, and across a gravelled walk to a smaller house within the enclosure of her own grounds, where, feeling a little tired, she had lain down upon a bed; that soon after she experienced slight discomfort, likened by her to ill-defined uneasiness of the abdomen under the operation of a mild laxative, followed by an impression that some solid warm body was lying in contact with her person; that she directed her servant to look below the bedclothes, and that the attendant, on doing so, found to her surprise the child entirely extruded.

"My patient assured me repeatedly and earnestly that she was quite unconscious of the whole parturient process culminating in the birth of the child, and expressed herself both surprised and alarmed at a delivery so painless and instantaneous. As she was daily, nay, hourly, expecting her delivery, it is but reasonable to suppose that she had been for some time acutely alive to the earliest intimations of commencing parturition, and it is surely remarkable that nothing occurred from which she could have suspected that the act had actually commenced. My patient had no object in deceiving me, and I am quite satisfied of the entire truthfulness of her often—to me—repeated statement.

"This case has a medico-legal significance, as well as a practical. If a female awake, in perfect health, in the exercise of sound reason, and hourly expecting her confinement, having no object for its concealment, but many reasons for its occurrence, being welcomed by her friends, can be the subject of painless, unconscious labor, preceded by no appreciable premonitory symptoms, and making itself known only when the extrusion of the child has been completed in the way described, how much more may we be inclined to yield belief to cases in which it has been averred that delivery has taken place during sleep, without waking the mother, and to others, in which it has been maintained that owing to the painlessness of the parturient process, the child's life has been lost by a fall on the ground, or by being engulfed in a latrine? The child was a female, small, but not much undersized. The mother's first labor—this was the second—was a normal one, accompanied by the usual signs, and extending over six hours in its duration."

—— Dr. Pattison stated that he had once attended a primiparous patient who suffered no pain at all during labor. He had not been summoned to the case, but happened to call at the time; the child was born quite easily, the patient only experiencing a feeling of pressure.

Dr. Wilson had once been called to see a woman who had been delivered without any pain, whilst she was walking about in the house; and he found the child lying on the floor with the umbilical cord torn across.

Dr. Cochrane thought that such a case as that related by Dr. Smith might more readily occur in a warm country with a relaxing climate. But he had himself seen a woman who had just been delivered of a child almost unconsciously as she was getting out of bed.

Dr. Andrew Balfour stated that he had attended, when in China, the wife of an engineer on board a steamer, who suffered from remittent fever in the eighth month of her pregnancy. The whole ovum in that case was expelled entire without any warning; and when he (Dr. B.) arrived and ruptured the sac, the fœtus was already dead.

Dr. Pattison said Dr. Thatcher used to tell his class of a case where he found the patient had been delivered of an entire ovum with unruptured membranes. Dr. T. had been summoned by the husband, who was in great dismay, because, as he averred, his wife had given birth to a "leg of mutton."

Dr. Alex. R. Simpson stated that Von Ritgen, the venerable professor of midwifery at Giessen, had told him, that in a long course of his practice he had met with no less than seventeen cases of labor where the patient had experienced none of the ordinary labor pains; and he (Professor Von Ritgen) had been led to form the conclusion that in perfectly natural labor, pain should not necessarily be experienced, and that we had come to regard pain as a natural and necessary concomitant of labor, merely because women were almost never in a perfectly healthy condition when we were summoned to aid them during parturition. He (Dr. A. R. S.) thought that if Professor Von Ritgen's position could be established—and the facilities of parturition among savages went far to prove its truth—then the objection sometimes made to the use of chloroform in labor, on the ground of its being contrary to nature, would be most completely done away with.—*Ed. Med. Journ., Nov.*, 1862.—*Cin. Lancet and Observer.*

